# Adult-Onset Nephrotic Syndrome and Optic Nerve Atrophy Associated With *NUP93* Mutation

**DOI:** 10.1016/j.xkme.2025.101114

**Published:** 2025-09-19

**Authors:** Marc Scheen, Grégoire Arnoux, Philippe Khau Van Kien, Thomas Rio Frio, Giorgio Enrico Bravetti, Jean-Louis Blouin, Thomas Ernandez, Solange Moll, Frédéric Masclaux, Sophie De Seigneux, Marc Abramowicz, Fadi Haidar

**Affiliations:** 1 Department of Nephrology and Renal Transplantation, Hôpitaux universitaires de Genève, Rue Gabrielle-Perret-Gentil 4, 1205 Genève, Switzerland; 2 Department of Pathology, Hôpitaux universitaires de Genève, Rue Gabrielle-Perret-Gentil 4, 1205 Genève, Switzerland; 3 Department of Genetics, Hôpitaux universitaires de Genève, Rue Gabrielle-Perret-Gentil 4, 1205 Genève, Switzerland; 4 Department of Ophthalmology, Hôpitaux universitaires de Genève, Rue Gabrielle-Perret-Gentil 4, 1205 Genève, Switzerland

**Keywords:** FSGS, genetics, nephrotic syndrome, NUP93, optic nerve atrophy

## Abstract

Focal segmental glomerulosclerosis (FSGS) represents one of the most common etiologies of nephrotic syndrome. In 10% to 20% of cases, it is associated with steroid resistance and has the potential to progress to kidney failure. Compound heterozygote or homozygote mutations in the *NUP93* gene have been associated with FSGS-related nephrotic syndrome. To the best of our knowledge, no case of *NUP93* related FSGS has presented in the literature with associated ophthalmological anomaly. In this report, we present the case of a 25-year-old White patient who presented with rapidly progressive chronic kidney disease, congenital bilateral optic nerve atrophy and steroid-resistant nephrotic syndrome. Kidney biopsy showed FSGS with more than 80% foot process effacement on electronic microscopy. Genetic testing by means of whole exome sequencing later showed the presence of biallelic class 4, likely pathogenic variants in the *NUP93* gene. Although we cannot exclude another cause, such as a genetic defect outside the genomic regions screened by our exome analyses, for the bilateral optic atrophy observed in our patient, this suggests that this association is not coincidental. Our case report highlights the importance of genetic testing in cases of steroid-resistant nephrotic syndrome that present with syndromic congenital features.

Nephrotic syndrome is defined by the presence of excessive proteinuria, exceeding 3.5 g/24 h, accompanied by hypoalbuminemia, edema, and hyperlipidemia.^1^^,^^2^ Focal segmental glomerulosclerosis (FSGS) represents one of the most common etiologies of nephrotic syndrome in both pediatric and adult patients.^3^ In 10% to 20% of cases, it is associated with steroid resistance and has the potential to progress to kidney failure.^4^, ^5^, ^6^

Primary FSGS cases are genetically heterogeneous disorders, representing a spectrum of hereditary kidney diseases.^3^^,^^7^ It can be inherited in an autosomal-dominant, autosomal-recessive (the most frequent form), or X-linked manner.^7^ Nephrotic syndrome type 12 is caused by a compound heterozygote or homozygote mutation in the *NUP93* gene.^8^ This gene encodes an ubiquitous nucleoporin protein, which functions in the active transport of molecules between the nucleus and cytoplasm.^7^ It has been demonstrated that mutations in *NUP93* are responsible for the development of nonsyndromic autosomal-recessive FSGS in children.^7^ In this case report, we present a patient with adult-onset steroid-resistant nephrotic syndrome and bilateral optic nerve atrophy. Genetic analysis showed a compound heterozygous mutation in *NUP93*, and functional analysis confirmed the effect of these variants on splicing. This presents an atypical phenotype in comparison with other cases of *NUP93* gene mutations.

## Case Presentation

A 25-year-old Maldovan patient with a history of bilateral optic nerve atrophy, bicuspid aortic valve, chronic gastritis, and osteochondritis dissecans was referred to the renal clinic in 2019 by his general practitioner. He has been under the care of the ophthalmology department since the age of 2, with a diagnosis of progressive bilateral optic atrophy of unknown origin. By the age of 21, the patient’s best-corrected visual acuity had already deteriorated to 0.6 in both eyes. Color vision was also significantly impaired, with Ishihara test results of 3/12 in the right eye and 2/13 in the left eye. Fundoscopic examination showed severe bilateral optic nerve pallor, predominantly affecting the temporal quadrants, with no additional retinal abnormalities. Optical coherence tomography of the retinal nerve fiber layer demonstrated marked bilateral atrophy, with a global thickness of 48 μm in the right eye and 49 μm in the left eye. Similarly, ganglion cell layer analysis showed a significant reduction in volume, measuring 0.72 mm^3^ in the right eye and 0.67 mm^3^ in the left eye. Visual field testing showed a generalized reduction as well in sensitivity, with multiple scotomas localized within the central 10 degrees. Whole exome sequencing conducted during his childhood did not show any pathological or even candidate variant in genes associated with optic nerve atrophy. He initially presented in early 2019 with an asymptomatic elevation in creatinine levels of unknown origin, with a measured value of 167 μmol/L (eGFR 50 mL/min/1.73 m^2^) and nephrotic range proteinuria of 6 g/2 4h, accompanied by hypoalbuminemia at 30 g/L. The patient had no documented family history of ophthalmological disease, hypertension, nephrotic syndrome, or chronic kidney disease.

The immunological panel, comprising C3, C4, anti-dsDNA, anti-neutrophil cytoplasmic antibodies, antinuclear antibodies, cryoglobulin, and serum protein electrophoresis, yielded unremarkable results. Viral serologies, including HBsAg, anti-HBs IgG, anti-HCV IgG and IgM, anti-gp124 IgG and IgM, and p24, were negative. Kidney ultrasound showed small kidneys, with lengths of 8.6 cm on the right and 8.5 cm on the left. Additionally, bilateral cortical atrophy and loss of corticomedullary differentiation were observed. However, Doppler imaging indicated preserved vascularization.

**Figure 1:**
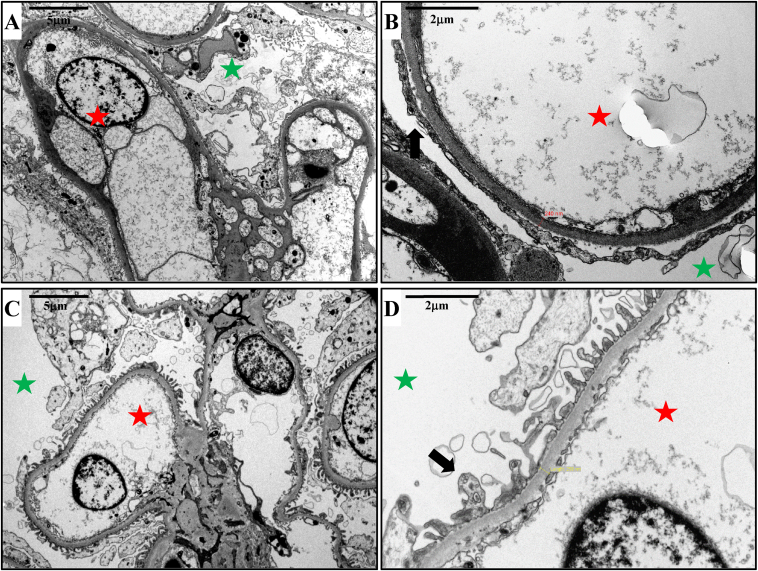
Electron microscopy findings: Electron microscopy analysis showed diffuse and severe podocyte foot process effacement involving more than 80% of the basement membrane’s surface area in nonsclerotic glomeruli, highly suggestive of a primary focal segmental glomerulosclerosis (FSGS) (A, B). Post-transplant follow-up at 3 years showed no disease recurrence (C, D). Green and red stars indicate respectively urinary and intracapillary spaces. Black arrow indicates podocyte foot process. Magnification scale: A and C, 5 μm ; B and D, 2 μm.

A kidney biopsy was subsequently conducted, which showed the presence of focal and segmental glomerulosclerosis, accompanied by mild interstitial nephritis and pronounced interstitial fibrosis, estimated at 70% of the cortical surface area. The diagnosis of primary FSGS was corroborated by electronic microscopy, which also confirmed extensive podocyte foot process effacement of more than 80% of the basement membrane’s surface area in nonsclerotic glomeruli (Fig 1).

The patient was initiated on a pulsed corticosteroid regimen, followed by a 1 mg/kg/day dose of prednisone for a period of 16 weeks. To reinforce the nephroprotective measures already in place, an angiotensin-converting enzyme inhibitor was added to the treatment plan, along with a recommendation for a low-salt diet. No improvement in proteinuria or kidney function was observed during the course of treatment, and the patient did not achieve any complete or partial remission on steroids. Subsequently, he exhibited signs of acquired secondary Cushing's syndrome, including weight gain and new-onset hypertension. The corticosteroids were gradually discontinued, and the decision was made not to switch to calcineurin inhibitor therapy because of extensive fibrosis observed in the initial kidney biopsy.

It is regrettable to report that the patient's kidney function declined markedly over a period of several months. He was admitted to the hospital on a provisional basis because of hypertensive urgency, which was a consequence of his failure to comply with the instructions provided regarding his antihypertensive medication. Subsequently, he developed kidney failure in early 2020, necessitating kidney replacement therapy via maintenance hemodialysis. During that period, the patient was assessed for the possibility of undergoing a kidney transplant at our institution. A potential donation was evaluated from the patient's mother.

**Figure 2:**
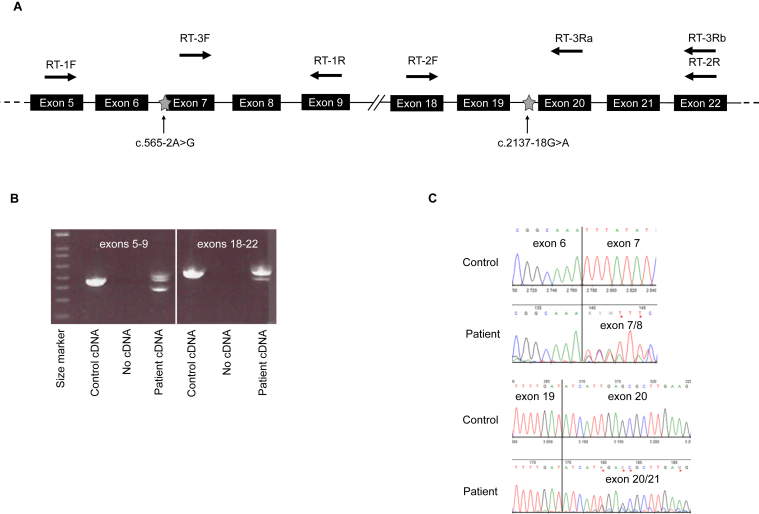
RNA study. (A) Schematic representation of part of the NUP93 gene. Stars indicate the position of the variants and arrows the location of the primers used for the different RT-PCR experiments (RT1, RT2, RT3a and b). (B) Effects on the splicing of variants detected in NUP93. Electrophoresis of the RT–PCR1 and 2 products obtained from the NUP93 transcript regions by RT-PCR1: exons 5 to 9 (left) and RT-PCR2: exons 18 to 22 (right), showing a second product in patient’s lane. (B) Sequencing traces of RT-PCR1 and 2 products as in B. RT-PCR, reverse transcription polymerase chain reaction.

A whole exome sequencing test was conducted as part of the pretransplant evaluation, given the suspicion of a monogenic form of FSGS associated with possible syndromic-like features of bilateral optic nerve atrophy and bicuspid aortic valve. Whole exome sequencing was conducted and analyzed using a specific combined panel of genes associated with renal, optic nerve atrophy, mitochondrial disorders, and unexplained kidney failure in young patients (Renome_332_v5, Optic_atrophy_21_v2, PanelApp/Unexplained_kidney_failure_in_young_people_v1.96, Panel App/Mitochondrial_disorders_v2.51) and then further extended to 3,935 genes involved in Mendelian diseases (PanelApp(Aus):Mendeliome (Version 1.1902): https://mcri-panelapp-test.org/panels/137/). This showed the presence of 2 heterozygous *NUP93* variants predicted to alter splicing *in silico*: NM_014669.5:c.565-2A>G(;)c.2137-18G>A. No other variant in genes related to optic atrophy or bicuspid aortic valve was observed. *NUP93* transcripts analyzes were then conducted from total RNA extracted from patient’s blood to evaluate the predicted effects on splicing. As illustrated in Fig 2, the variant c.565-2A>G results in the skipping of exon 7, leading to an in-frame deletion of 30 amino acids, from positions 189 to 218 of the protein. The variant c.2137-18G>A was identified as an hypomorphic allele, as it partially skips exon 20 at the mRNA level and is predicted to produce both a normal protein and a variant with an in-frame deletion of 28 amino acids from positions 713 to 740. In silico structural analysis of the abnormal proteins^9^ predicted that these deletions disrupt the protein structure by causing a loss of alpha helices. Only the patient’s mother was available to study parental segregation of each variant. Study of DNA extract from her blood sample shown the presence of the c.565-2A>G variant. To determine the phase of the 2 variants in the patient, NUP93 mRNA spanning exons 7 to 22 was amplified using reverse transcription polymerase chain reaction. A forward primer located in exon 7 was used to ensure that transcripts harboring the c.562-2A>G variant (and thus lacking exon 7) were not amplified. Exon 20 skipping was detected in the resulting PCR product, indicating that the c.2137-18G>A variant resides on a different transcript from c.562-2A>G. This confirmed that the 2 variants were in trans. According to the standards and guidelines for the interpretation of sequence variants of the American College of Medical Genetics,^10^ both variants were classified as likely pathogenic, class 4, explaining most likely the patient’s phenotype.

Subsequently, the patient underwent a living kidney transplantation from his mother. The patient is currently at 40 months post-transplantation with no documented relapse of the disease. In addition, kidney allograft biopsy was performed after 36 months for suspected rejection and no disease relapse was histologically observed on the allograft.

## Discussion

The present case report describes a unique instance of adult-onset, rapidly progressive chronic kidney disease (CKD) with nephrotic range proteinuria and syndromic-like features. These manifestations were caused by steroid-resistant primary FSGS related to variants altering *NUP93* functions. The case is further distinguished by the unreported association of congenital bilateral optic nerve atrophy with adult-onset steroid-resistant FSGS.

The association between steroid-resistant primary FSGS and *NUP93* pathogenic variants has been documented by several authors.^7^^,^^8^^,^^11^ These patients typically have a poor prognosis, with rapid disease progression to kidney failure within 10 years. The documented pathogenic variants have been shown to be associated with early-onset nephrotic syndrome, typically occurring during the first decade of life. To date, there have been no published adult-onset cases. Although the in trans combination of a pathogenic variant and a hypomorphic variant is consistent with the recessive inheritance pattern of *NUP93*-related FSGS, the production of a limited amount of normal NUP93 protein from the hypomorphic allele may have significantly contributed to the delayed onset of the disease. Furthermore, our patient progressed to kidney failure faster than the published cases, with an onset of renal replacement therapy occurring within 5 years of diagnosis.

One case of FSGS was attributed to a compound heterozygous variants in the *NUP93* gene by Rossanti et al.^12^ To the best of our knowledge, no single or compound variants of *NUP93* have been associated with both steroid-resistant primary FSGS and bilateral optic atrophy.

NUP93 is a ubiquitous nuclear pore protein that serves as a key structural component of the nuclear pore complex. In the kidney, it appears to be involved in the regulation of podocyte proliferation. A mutation in *NUP93* results in the inhibition of podocyte proliferation because of a defect in supressor of mother against decapentaplegic familly proteins signaling. It plays a pivotal role in the functioning of cilia as well as in myocardial embryogenesis. The abnormal NUP93 proteins produced by the patient's cells have an altered scaffold structure of alpha helices because of deletions observed at the mRNA level. These deletions are highly likely to affect the interaction of NUP93 with its numerous binding partners, ultimately affecting the efficiency of nuclear pore complex assembly and/or stability.^13^^,^^14^ This hypothesis is further supported by previous reports of an in-frame deletion of exon 13 at the mRNA level, caused by a splicing mutation, in which the resulting protein was shown to compromise nuclear pore complex integrity.^15^

Other mutations in *NUP93* have been associated with nonprogressive congenital ataxia^16^ and the nucleoporin gene have been linked to the onset of dilated cardiomyopathy.^17^ Although we cannot exclude another cause, such as a genetic defect outside the genomic regions screened by our exome analyses, for the bilateral optic nerve atrophy observed in our patient, our findings suggest that this association is unlikely to be coincidental.

In the case of kidney disease associated with *NUP93*, it seems therefore important to look for other abnormalities such as neurological features to answer the question of the phenotypic spectrum of *NUP93* mutations.
